# Virulence, Antimicrobial Resistance Properties and Phylogenetic Background of Non-H7 Enteropathogenic *Escherichia coli* O157

**DOI:** 10.3389/fmicb.2016.01540

**Published:** 2016-09-28

**Authors:** Mithila Ferdous, Anna M. D. Kooistra-Smid, Kai Zhou, John W. A. Rossen, Alexander W. Friedrich

**Affiliations:** ^1^Department of Medical Microbiology, University Medical Center Groningen, University of GroningenGroningen, Netherlands; ^2^Department of Medical Microbiology, Certe Laboratory for Infectious DiseasesGroningen, Netherlands; ^3^State Key Laboratory for Diagnosis and Treatment of Infectious Diseases, Collaborative Innovation Centre for Diagnosis and Treatment of Infectious Diseases, The First Affiliated Hospital, School of Medicine, Zhejiang UniversityHangzhou, China

**Keywords:** enteropathogenic *Escherichia coli* (EPEC), antimicrobial resistance, virulence, mobile genetic elements, whole genome sequencing, phylogenetic relationship, evolution

## Abstract

*Escherichia coli* (*E*.coli) O157 that do not produce Shiga toxin and do not possess flagellar antigen H7 are of diverse H serotypes. In this study, the antibiotic resistance properties, genotype of a set of virulence associated genes and the phylogenetic background of *E. coli* O157:non-H7 groups were compared. Whole genome sequencing was performed on fourteen O157:non-H7 isolates collected in the STEC-ID-net study. The genomes were compared with *E. coli* O157 genomes and a typical Enteropathogenic *E. coli* (tEPEC) genome downloaded from NCBI. Twenty-six (86%) of the analyzed genomes had the intimin encoding gene *eae* but of different types mostly correlating with their H types, e.g., H16, H26, H39, and H45 carried intimin type ε, β, κ, and α, respectively. They belonged to several *E. coli* phylogenetic groups, i.e., to phylogenetic group A, B1, B2, and D. Seven (50%) of our collected O157:non-H7 isolates were resistant to two or more antibiotics. Several mobile genetic elements, such as plasmids, insertion elements, and pathogenicity islands, carrying a set of virulence and resistance genes were found in the *E. coli* O157:non-H7 isolates. Core genome phylogenetic analysis showed that O157:non-H7 isolates probably evolved from different phylogenetic lineages and were distantly related to the *E. coli* O157:H7 lineage. We hypothesize that independent acquisition of mobile genetic elements by isolates of different lineages have contributed to the different molecular features of the O157:non-H7 strains. Although distantly related to the STEC O157, *E. coli* O157:non-H7 isolates from multiple genetic background could be considered as pathogen of concern for their diverse virulence and antibiotic resistance properties.

## Introduction

Enteropathogenic *Escherichia coli* (EPEC) was first identified in the United Kingdom in the 1940s as the cause of outbreaks of infantile diarrhea (Bray, 1945). Although such outbreaks are now rare in developed countries, EPEC strains continue to be a leading cause of diarrhea among infants from developing countries (Chen and Frankel, 2005). The most important feature of EPEC pathogenesis is its ability to produce characteristic histopathological intestinal lesions known as “attaching and effacing” (A/E) lesions that are also characteristic for some Shiga toxin-producing *E. coli* (STEC) (Jerse et al., 1990). The genes responsible for this activity in EPEC are encoded on a 35-kb pathogenicity island (PAI) called the locus of enterocyte effacement (LEE), whereas the LEE of STEC contains some additional genes encoded within a putative prophage designated 933L (Perna et al., 1998). Both EPEC and STEC LEE encode a type III secretion system, multiple secreted proteins, and a bacterial adhesin called intimin (Nataro and Kaper, 1998). The 5′ regions of intimin-encoding gene *eae* are conserved in both EPEC and STEC, whereas the 3′ regions are heterogeneous even within the same pathotype (Blanco et al., 2004). Several variants of the *eae* gene encoding different intimin types and subtypes are described (Oswald et al., 2000; Zhang et al., 2002; Blanco et al., 2006) and it has been suggested that different intimins may be responsible for different host tissue cell tropisms (Torres et al., 2005).

EPEC, carrying EPEC adherence factor plasmid (pEAF) is named as typical EPEC (tEPEC), while those without the pEAF as atypical EPEC (aEPEC) (Trabulsi et al., 2002; Hernandes et al., 2009). aEPEC are thought to be a concern as they can acquire Shiga toxin (Stx) converting bacteriophages, thereby obtaining the ability to cause more serious illness (Bolton et al., 2014). Besides the virulence genes encoded on LEE, EPEC strains also carry other virulence genes including *astA* (heat stable enterotoxin), the *cdt* (cytolethal distending toxin) gene cluster, *efa*1 (enterohemorrhagic *E. coli* factor for adherence), and *paa* (porcine attaching-effacing associated protein) (Bouzari and Varghese, 1990; Badea et al., 2003; Batisson et al., 2003; Dulguer et al., 2003).

Antibiotic resistance is a global concern due to the increased use of antibiotics, especially for the intestinal organisms as the gut is a heavily populated niche and resistance genes can be transmitted horizontally via these resistant organisms in the gut (Scaletsky et al., 2010). High prevalence of antimicrobial resistance among EPEC strains has been reported in different countries but the genetic basis for this resistance and the evolutionary consequences are rarely studied (Senerwa et al., 1991; Abe et al., 2009; Medina et al., 2011).

The serotyping of O antigens (together with the H-flagellar antigen) is used as an effective method to identify various pathogenic clones (Iguchi et al., 2011). The serotype O157 is a predominant serotype of the documented STEC related outbreaks worldwide (Nataro and Kaper, 1998; Caprioli et al., 2005) and is frequently associated with the H7 antigen (encoded by *fliC* H7). *E. coli* O157:H7 strains that do not possess Stx are presumably the Stx-lost variants or progenitors of STEC as they share the same phylogenetic lineage (Ferdous et al., 2015). However, the large and diverse O157 serogroup also includes many non-H7 serotypes that are commonly found in animals, food, and clinical samples. EPEC strains of the O157:H45 serotype have been noted as agents of diarrhea in outbreaks and in sporadic cases in Germany, Japan, Korea, and Thailand (Blank et al., 2003; Park et al., 2014). Strains of serotype O157:H8 and O157:H16 have been isolated from cases of diarrhea in human, whereas the latter serotype was also isolated from cattle, beef, and water (Feng et al., 2010; Iguchi et al., 2011). As these strains do not carry the *stx* gene, they are often not detected or no detailed characterization is carried out (Feng et al., 2010). Recently, two studies were performed on comparative genomics analysis and phylogenetic relationship of *E. coli* O157:H7 and non-H7 strains (Sanjar et al., 2015; Kossow et al., 2016). However, there is a lack of information regarding their virulence, antimicrobial resistance properties and some other molecular features. In our current study, apart from the phylogenetic and evolutionary relationship of *E. coli* O157:non-H7 groups, a comprehensive characterization was performed focusing on their diversity in virulence and antimicrobial resistance properties.

## Materials and methods

### Selection of isolates for the study

A total of 14 *E. coli* O157 strains that were negative for *fli*C H7 gene were selected for this study. Isolates were obtained from fecal samples of patients with gastrointestinal complaints in the regions of Groningen and Rotterdam during the period April 2013–March 2014, as part of a large multicenter study (STEC-ID-net, unpublished data).

Additionally, publically available genomes of 16 *E. coli* including two O157:H7, one O55:H7, 12 O157:non-H7 and one tEPEC strain (E2348/69; O127:H6) were included in the comparative analyses. The information of isolates and downloaded genomes used in this study are presented in Table 1.

**Table 1:** *****E. coli*** isolates used in this study**.

**Isolate ID**	**Isolation region^a^**	**Source**	**Symptoms^b^**	**Genoserotype**	**MLST**	**Phylogenetic group**	**Intimin type**	**Accession No**.	**References**
EPEC 400	Groningen (NL)	Human	D	O157:H16	10	A	ε	LZDU00000000	This study
EPEC 536	Groningen (NL)	Human	ND	O157:H16	10	A	ε	LZDV00000000	This study
EPEC 631	Groningen (NL)	Human	N	O157:H16	10	A	ε	LZDW00000000	This study
EPEC 720	Groningen (NL)	Human	N	O157:H16	10	A	ε	LZDX00000000	This study
EPEC 1316	Groningen (NL)	Human	D	O157:H16	10	A	ε	LZDY00000000	This study
EPEC 2646	Rotterdam (NL)	Human	N	O157:H16	10	A	ε	LZDZ00000000	This study
EPEC 2669	Rotterdam (NL)	Human	N	O157:H16	10	A	ε	LZEA00000000	This study
EPEC 3029	Rotterdam (NL)	Human	N	O157:H16	10	A	ε	LZEB00000000	This study
EPEC 1150	Groningen (NL)	Human	D	O157:H39	4554	B2	κ	LZEC00000000	This study
EPEC 1554	Groningen (NL)	Human	D	O157:H39	4554	B2	κ	LZED00000000	This study
EPEC 2252	Rotterdam (NL)	Human	D	O157:H39	4554	B2	κ	LZEE00000000	This study
EPEC 2272	Rotterdam (NL)	Human	D	O157:H39	4554	B2	κ	LZEF00000000	This study
EPEC 2081	Rotterdam (NL)	Human	N	O157:H26	189	A	β	LZEG00000000	This study
EPEC 2827	Rotterdam (NL)	Human	D	O157:H26	189	A	β	LZEH00000000	This study
Sakai	Japan	Human	HUS	O157:H7	11	D	γ	NC_002695	Hayashi et al., 2001
SS52	USA	Cattle	NA	O157:H7	11	D	γ	CP010304	Katani et al., 2015
CB9615	Germany	Human	D	O55:H7	335	D	γ	CP001846	Zhou et al., 2010
Santai	China	Duck	NA	O157:H16	1011	D	NA	CP007592	Cheng et al., unpublished
3006	USA	Human	N	O157:H16	5502	A	NA	AMUN01000000	Sanjar et al., 2015
TW15901	France	Food	NA	O157:H16	10	A	ε	AMUK01000000	Sanjar et al., 2015
TW00353	USA	Human	N	O157:H16	10	A	ε	AMUM01000000	Sanjar et al., 2015
C639_08	Denmark	Human	N	O157:H45	725	B2	α	AIBH00000000	Hazen et al., 2013
C844_97	Japan	Human	N	O157:H45	725	B2	α	AIBZ01000000	Hazen et al., 2013
RN587/1	Brazil	Human	N	O157:H45	725	B2	α	ADUS01000000	Hazen et al., 2013
ARS4.2123	USA	Water	NA	O157:H45	725	B2	α	AMUL01000000	Sanjar et al., 2015
TW07793	Unknown	Water	NA	O157:H39	1041	B2	κ	AFAG0200000	Sanka et al., unpublished
7798	Argentina	Human	N	O157:H39	5611	B2	κ	AMUP00000000	Sanjar et al., 2015
N1	Unknown	Food	NA	O157:H29	515	B1	NA	AMUQ01000000	Sanjar et al., 2015
T22	Hungary	Human	N	O157:H43	155	B2	NA	AHZD02000000	Sanjar et al., 2015
E2348/69	Taunton, United Kingdom	Human	D	O127:H6	15	B2	α	FM180568	Iguchi et al., 2009

a *NL, the Netherlands*.

b *D, Diarrhea; ND, Abdominal pain and other gastrointestinal problems without diarrhea; N, Not available; HUS, Hemolytic uremic syndrome; NA, Not applicable*.

### Phenotypic characterization and antibiotic resistance profile

Sorbitol fermentation was determined using CT-SMAC plates (sorbitol MacConkey agar with cefixime and tellurite, Mediaproducts BV, Groningen, the Netherlands). Motility was tested using Motility test medium with triphenyltetrazolium chloride (Mediaproducts BV, Groningen, the Netherlands). The production of beta-glucuronidase and urease were checked by using MacConkey II agar with 4-methylumbelliferryl-β-D-glucuronide (MUG) (BD Diagnostics, Breda, the Netherlands) and urea-triple sugar iron (TSI) agar (Mediaproducts BV, Groningen, the Netherlands), respectively. The O and H serotypes of the isolates were determined by seroagglutination performed at the National Institute for Public Health and the Environment (RIVM, Bilthoven, the Netherlands). Antibiotic resistance patterns of the isolates were determined using VITEK2 (bioMérieux, Marcy l'Etoile, France) following EUCAST guidelines.

### Whole genome sequencing

From all the isolates DNA was extracted using the UltraClean® microbial DNA isolation kit (MO BIO Laboratories, Carlsbad, CA, US) according to the manufacturer's protocol. A DNA library was prepared using the Nextera XT kit (Illumina, San Diego, CA, US) according to the manufacturer's instructions and then ran on a Miseq (Illumina) for generating paired-end 250-bp reads aiming at a coverage of at least 60 fold as described previously (Ferdous et al., 2015; Zhou et al., 2015).

### Data analysis and molecular typing

*De novo* assembly was performed using CLC Genomics Workbench v7.0.3 (CLC bio A/S, Aarhus, Denmark) after quality trimming (Qs ≥ 28) with optimal word sizes based on the maximum N50 value (Ferdous et al., 2015; Zhou et al., 2015). All the assembled genomes of this study and the assembled genomes downloaded from NCBI were subjected to further analyses. Annotation was performed by uploading the assembled genomes onto the RAST server version 2.0 (Aziz et al., 2008). The sequence type (ST) was identified by uploading the assembled genomes to the Center for Genomic Epidemiology (CGE) MLST finder website (version 1.7) (Larsen et al., 2012). For four of the isolates the allele numbers were submitted to the *E. coli* MLST databases (http://mlst.warwick.ac.uk/mlst/dbs/Ecoli) to obtain a ST for them. The virulence genes were determined by virulence finder 1.2 (Joensen et al., 2014), antibiotic resistance genes were determined by ResFinder 2.1 (Zankari et al., 2012) and the serotyping genes of the isolates were confirmed using the SeroTypeFinder tool (Joensen et al., 2015) from the CGE server. Intimin types of the isolates were determined using blastn. Isolates were assigned to one of the major *E. coli* phylogenetic groups A, B1, B2, or D using the genetic markers *chuA, yjaA*, and the DNA fragment TspE4.C2 (Clermont et al., 2000).

### Synteny analysis of resistance and virulence genes

To determine the location of virulence and antibiotic resistance genes in the isolates, the contigs containing the targeted genes were subjected to BLAST in NCBI to look for the most related genomic regions which were later used as reference to confirm their presence in our analyzed isolates.

To compare the sequences of the LEE region, the LEE of the *E. coli* O157:H7 strain 71074 (accession no GQ338312) was used as the reference and the contigs of each sample were subjected to BLAST against the reference and plotted by BLAST ring image generator (BRIG) (Alikhan et al., 2011).

### Phylogenetic analysis

To determine the phylogenetic relationship of the isolates, a gene-by-gene approach was performed using SeqSphere^+^ v3.0 (Ridom GmbH, Münster, Germany). For this, an *ad-hoc* core genome MLST (cgMLST) scheme was used as described previously (Ferdous et al., 2016). Briefly, the genome of *E. coli* O157:H7 strain Sakai was taken as a reference genome and ten additional *E. coli* genomes were used as query genomes to extract open reading frames (ORFs) using MLST+ Target Definer 2.1.0 of SeqSphere^+^. Only the ORFs without premature stop codon and ambiguous nucleotides from contigs of assembled genomes were included. The genes shared by the genomes of all isolates analyzed in this study were defined as the core genome for phylogenetic analysis (Ferdous et al., 2016). A Neighbor Joining (NJ) tree was constructed based on a distance matrix among differences in the core genome of the isolates.

## Results

### Phenotype

Ten of the fourteen O157 isolates collected in our study were motile and were of serotype O157:H16 (*n* = 8) or O157:H26 (*n* = 2); the other four were non-motile. All the isolates were positive for beta-glucuronidase and negative for urease. Except two isolates (EPEC 2646 and EPEC 2669) all grew on CT-SMAC agar and fermented sorbitol.

### Molecular typing

The molecular typing results of the *E. coli* O157 isolates included in this study are summarized in Table 1. The four non-motile isolates of this study were confirmed to carry the *fliC* H39 gene and were assigned to a new ST (ST 4554). All of the O157:H16 isolates belonged to *E. coli* phylogenetic group A except strain Santai which belonged to phylogenetic group D (phylogenetic group of STEC O157:H7). None of the strains were positive for the *stx* gene.

### Virulence profiling and intimin typing

**Figure 1:**
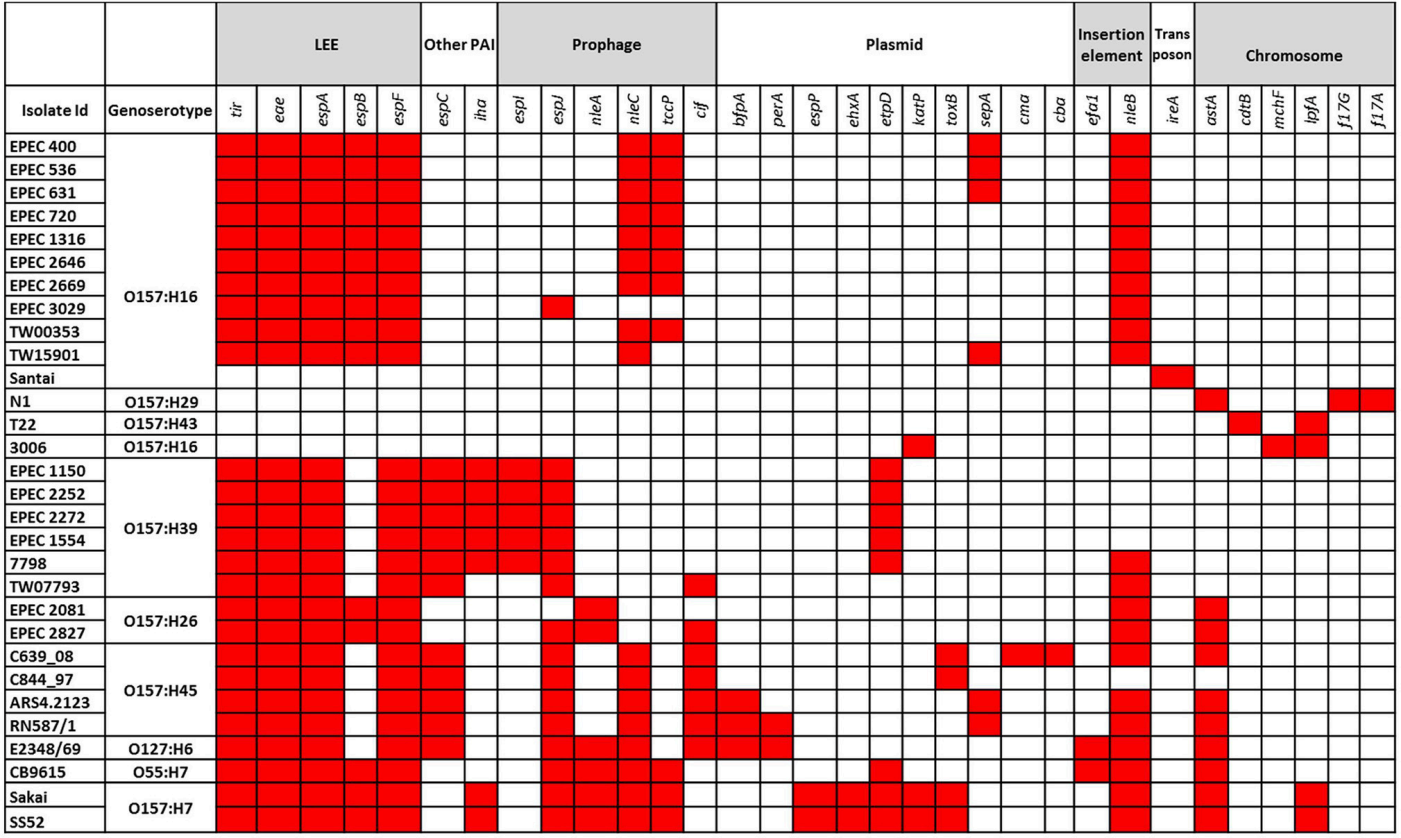
**Virulence profiles of the isolates analyzed in this study**. Predicted locations of the virulence genes are indicated. The red color indicates the presence of a gene in the corresponding isolate.

The virulence profiles of the isolates and of the genomes are presented in Figure 1. Four of the genomes obtained from NCBI (N1, T22, 3006, and Santai) did not have the *eae* gene. The rest carried the gene but different types of it were found mostly correlating to their genoserotypes, e.g., O157:H16 and O157:H39 isolates carried intimin type ε and κ respectively (Table 1). None of the isolates carried intimin γ. Like tEPEC E2348/69, all O157:H45 strains had intimin α.

Besides the virulence genes located on the LEE PAI, isolates contained other virulence genes carried on several mobile genetic elements (MGEs), e.g., the plasmid-borne genes *etpD, toxB, sepA*, the prophage encoded genes *espI, tccP, cif*, the PAI related genes *iha* and *espC*, and other virulence determinants carried on insertion elements or transposons (Figure 1). All O157:H39 isolates carried the *etpD* gene located on pO55 like (O55:H7 strain CB9615) plasmid encoding the type II secretion system proteins (*etp* gene cluster) and the conjugal transfer proteins (*tra* gene cluster) (Supplementary Figure S1), and adhesion gene *iha* presumably on a transposon. All of the O157:H45 and O157:H39 strains carried the *espC* PAI (Supplementary Figure S2). All the isolates analyzed in this study were negative for the pillin subunit encoding gene *sfp*A.

### Comparison of LEE

**Figure 2:**
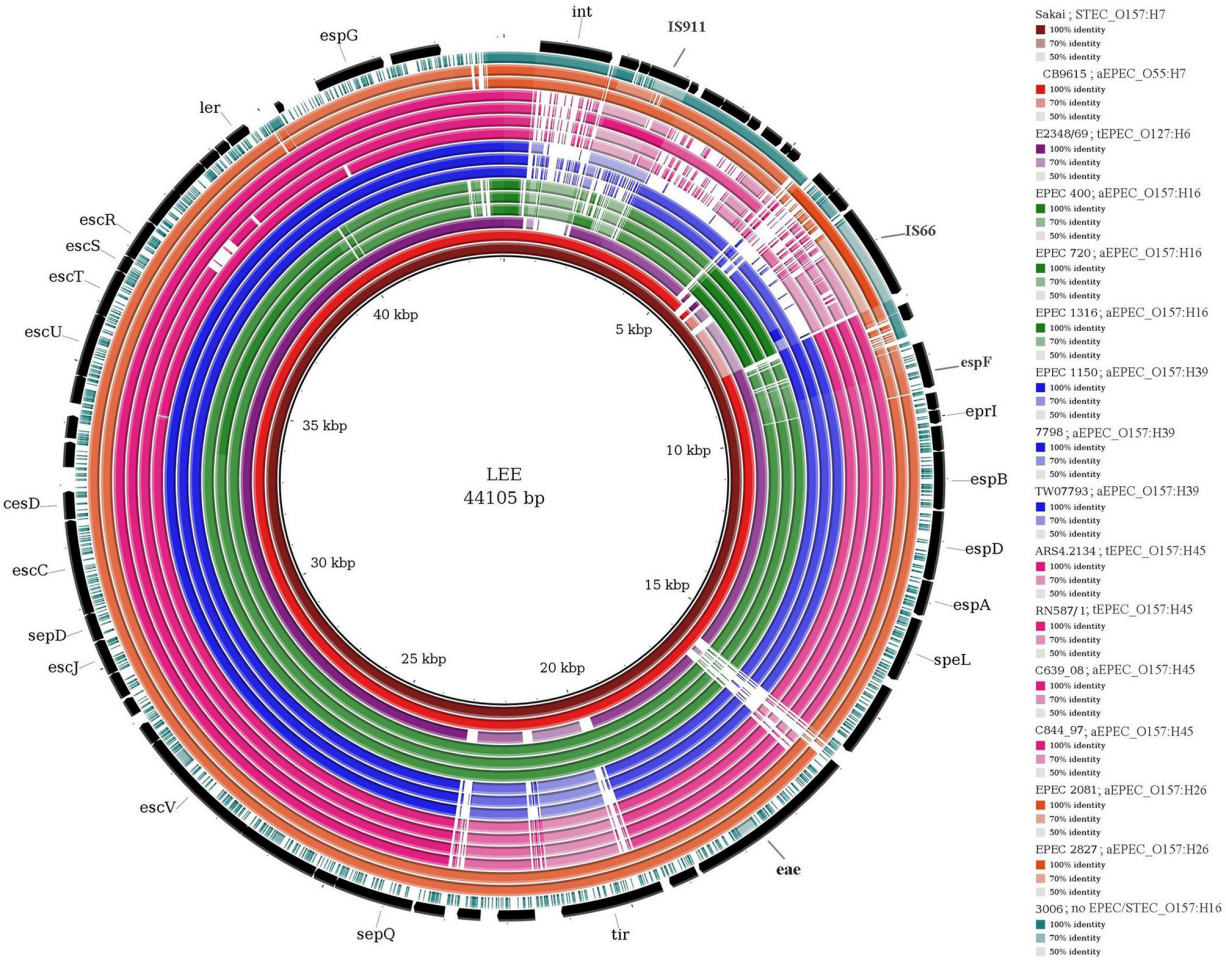
**Comparison of LEE in ***E. coli*** isolates analyzed in this study**. The figure shows BLAST comparison of *E. coli* isolates against the reference LEE sequence (core black circle). Each ring represents one isolate, different colors of the rings represent different genoserotypes. The gradients (dark, pale, and white) of each color represent the sequence similarity (from 100 to 0%) between samples and reference. The colors of different isolates as well as the order of the rings (from inner to outer) with the color gradient for sequence identity are shown in the legend (right). Please note that, for each genoserotype only representative isolates showing variations in the LEE sequences are presented in the figure.

Figure 2 shows the variations in the sequence of the LEE PAI in EPEC O157 isolates with different H types. The first 8 kb region, encoding the phage associated integrase and IS elements in STEC LEE, is not intact in EPEC isolates and some of them only contain part of it. For example, EPEC O157:H16 and EPEC 2827 isolates contain the insertion element IS66 and O157:H26 isolates contain a similar but not identical phage integrase as present in the STEC LEE. Among the core LEE genes, EPEC secreted proteins encoding genes (*esp* genes) and the genes encoding the proteins for adhesion (e.g., *eae, tir-*encoding translocated intimin receptor) of the EPEC isolates are different from those of STEC. Other genes encoding type III secretion system (e.g., *esc* and *sep* genes) and the regulator gene *ler* are similar in O157:H39 and O157:H45 EPEC isolates (represented by dark blue and dark pink color in Figure 2, respectively) to those of STEC but are different in O157:H16 and O157:H26 (represented by the relatively light green and orange colors in Figure 2, respectively). The sequence of the *tir* gene is conserved between O157:H16 and O157:H26 serotypes (97–98% similar), and between O157:H39 and O157:H45 serotypes (98–99% similar). Notably, the *tir* gene of H39 and H45 is similar (97–98%) to that of tEPEC E2348/69, whereas in H16 and H26 it is different (<78% similarity) from all other serotypes analyzed in this study (data not shown). Clearly, the sequence of the LEE region is variable in different O157 EPEC strains.

### Antibiotic resistance profile and presence of resistance genes

**Figure 3:**
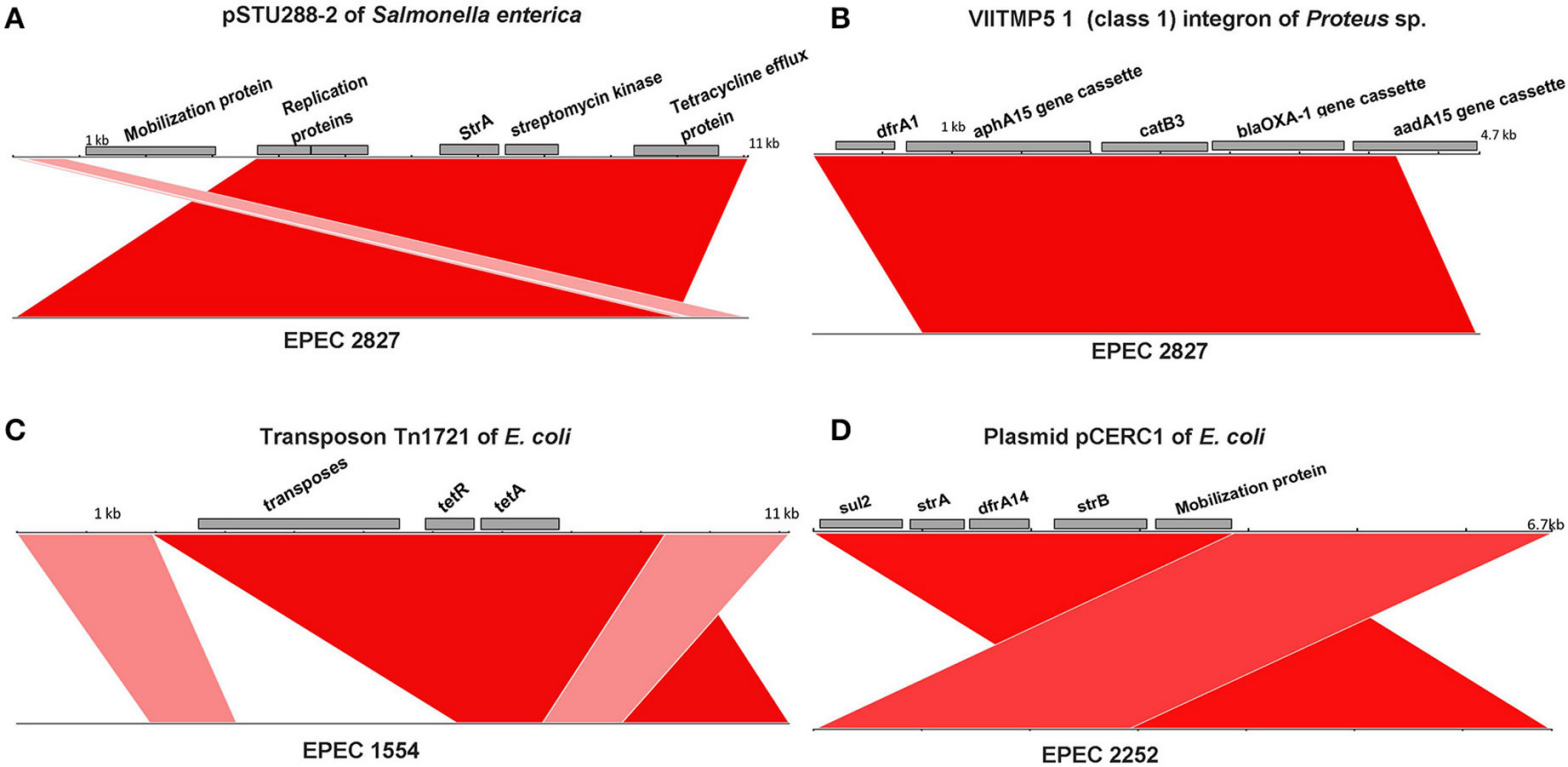
**Comparison of mobile genetic elements containing antibiotic resistance genes**. Resistance genes of EPEC 2827 are carried on a plasmid similar to pSTU288-2 of *Salmonella enterica* **(A)** and on an integron similar to VIITMP5 1 of *Proteus* sp. **(B)**. **(C)** Presence of a transposon like Tn1721 in EPEC 1554. **(D)** Presence of plasmid like pCERC1 in O157:H39 strains is shown by a representative isolate (EPEC 2252). For all the figures, the color represents sequence identity on a sliding scale, the lighter the color, the lower the percentage identity.

Among the fourteen O157 isolates collected in this study, all O157:H39 and O157:H26 isolates and one of the O157:H16 isolates (EPEC 1316) were resistant to two or more antibiotics and EPEC 2827 was resistant to several antibiotics. The presence of antibiotic resistance genes was determined for all the genomes analyzed in this study (including those downloaded from NCBI without available phenotypic data) and the results are presented in Table 2. More than ten resistance genes were found in two isolates, EPEC 2827 and Santai. EPEC 2827 carried the resistance gene *str*A on a plasmid similar to pSTU288 (accession no. CP004059) of *Salmonella enterica* (Figure 3A), and *dfr*A, *aph*A, *aad*A, *bla*OXA, and *cat*B on an integron (accession no. HQ386835) of *Proteus* sp. VIITMP5 (Figure 3B). EPEC 1554 carried the *tet*A gene on a transposon similar to Tn1721 (accession no. X61367) (Figure 3C). The O157:H39 strains had a plasmid similar to pCERC1 (accession no. JN012467) of *E. coli* strain S1.2.T2R carrying a cluster of antibiotic resistance genes including *str*A-B, *sul*2, and *dfr*A14 (Figure 3D). EPEC 1316 carried the *str*A and *bla*TEM genes on a plasmid similar to pVR50A (accession no. CP011135) (data not shown) of *E. coli* VR50 caused an asymptomatic bacteriuria.

**Table 2:** **Antibiotic resistance profiles of the analyzed isolates**.

**Isolate ID^a^**	**Genoserotype**	**Phenotypical resistance^b^**	**Presence of Resistance genes^c^**
EPEC 1316	O157:H16	AMP, SXT, TMP	*blaTEM-1B, dfrA8, strA/B, sul2*
EPEC 1150	O157:H39	AMP, SXT, TMP	*blaTEM-1B, dfrA14, strA/B, sul2*
EPEC 2252	O157:H39	AMC, AMP, SXT, TMP	*blaTEM-1B, dfrA14, strA/B, sul2*
EPEC 2272	O157:H39	AMC, AMP, SXT, TMP	*blaTEM-1B, dfrA14, strA/B, sul2*
EPEC 1554	O157:H39	AMP, TET	*blaTEM-1B, tetA*
EPEC 2081	O157:H26	AMP, NOR, TET, TMP	*blaTEM-1B, dfrA8, tetA*,
EPEC 2827	O157:H26	AMP, CIP, FOX, GEN, NIT, NOR, SXT, TMP, TET, TOB	*aadB, aph(3′) IIA/XV, blaOXA-4, blaTEM-1B, catB, dfrA1, mphA, strA/B, sul2, tetA*
C639_08	O157:H45	NT	*sul2*
RN587/1	O157:H45	NT	*blaTEM-116*
Santai	O157:H16	NT	*aac(3)-IId, aac(6′)Ib-cr, aadA, armA, ARR-3, strA/B, blaOXA-1, blaTEM-1B, catA/B, dfrA12, floR, fosA, mphA/E, msrE, sul1/2, tetA*
TW00353	O157:H16	NT	*blaTEM-1C*
ARS4.2123	O157:H45	NT	*strA/B, sul2*
E2348/69	O127:H6	NT	*strA/B, sul2*

a *The first seven rows shaded gray are isolates collected in our study and their resistance profile was determined using VITEK2*.

b *AMP, ampicillin; AMC, amoxicillin-clavulanic acid; FOX, cefoxitin; CIP, ciprofloxacin; GEN, gentamicin; NIT, nitrofurantoin; NOR, norfloxacin; TET, tetracycline; TOB, tobramycin; TMP, trimethoprim; SXT, trimethoprim-sulfamethoxazole. NT, Not tested as only the genomes of these strains were available*.

c *Resistance genes mentioned in the table confer resistance to antibiotics of the following categories: aac(3)-IId, aac(6′)Ib-cr, aadA, aph(3′), arma, strA/B against aminoglycosides; ARR-3 against rifampicin; blaOXA, blaTEM against beta lactum antibiotics; catA/B, floR against phenicols; fosA against fosfomycin; mphA/E, msrE against macrolides; dfrA against trimethoprim; sul1/2 against sulfonamides; tet against tetracycline*.

### Phylogenetic analysis

**Figure 4:**
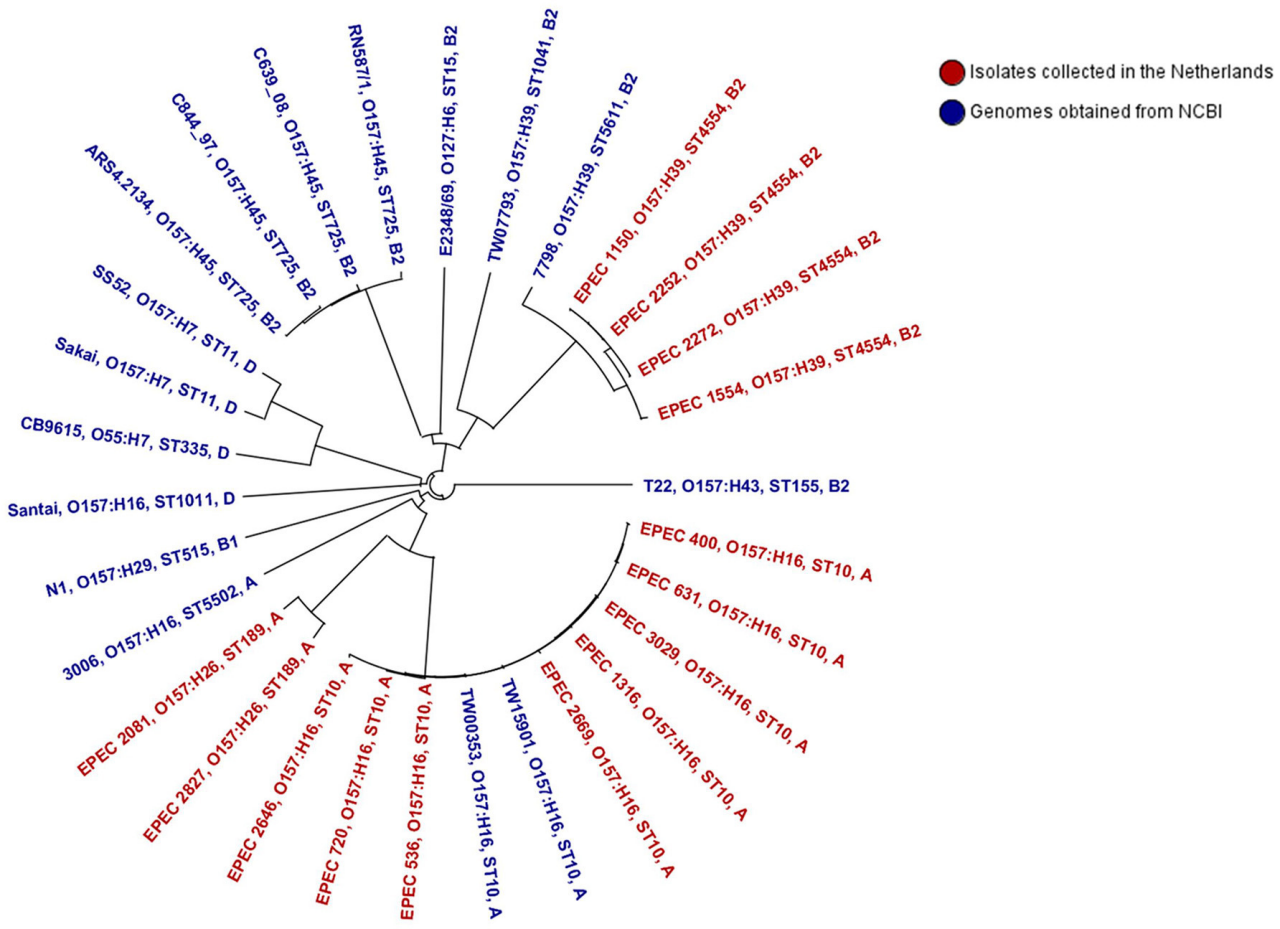
**Phylogenetic relationship of the isolates analyzed in this study**. This Neighbor Joining tree was constructed based on a distance matrix among differences in the core genome of the isolates. Each isolate Id is followed by its genoserotype, sequence type, and major phylogenetic group.

Figure 4 shows a NJ tree based on 2683 genes (core genome). The tree shows that strain Santai (phylogenetic group D) shared a common ancestor with the *E. coli* O157:H7 lineage and its progenitor O55:H7. The tEPEC O157:H45 (RN587/1 and ARS4.2123) and the aEPEC O157:H45 (C639_08 and C844_97) genomes clustered together and O157:H45 was the serogroup most closely related to tEPEC reference strain E2348/69. O157:H39 isolates were more closely related with tEPEC isolates than the O157:H16 and O157:H26 isolates. Three of the O157:H39 isolates (EPEC 1150, EPEC 2252, and EPEC 2272) appeared to be exactly similar in their core genome. Remarkably, except strain 3006 and Santai, the other ten O157:H16 isolates belonging to ST10 were closely related to each other with allele differences ranging from 0 to 41, whereas Santai and 3006 had a minimum of 2176 allele differences (data not shown) with the other O157:H16 isolates of this study indicating that only one fifth of their core genome contains identical alleles.

## Discussion

*E. coli* O157 strains are mostly known as enterohemorrhagic *E. coli* (EHEC) that have been associated with food born outbreaks worldwide. But they are also comprised of other *E. coli* pathogroups, including ones containing *eae* and lacking *stx*, which are considered as EPEC. *E. coli* O157:H7 isolates lacking *stx* gene are assumed to be members of STEC that lost the Stx encoding bacteriophages (Ferdous et al., 2015). The current study was performed to compare the molecular features and virulence properties of EPEC O157 having different H antigens (other than H7) with those of STEC O157:H7 isolates and to determine their phylogenetic relationship. STEC O157 isolates generally belong to ST11 and carry the *fliC* H7 gene encoding flagellar antigen H7 but the EPEC O157 isolates appeared to be from different sequence and H serotypes. The *stx* negative O157 isolates in this study were usually sorbitol fermenting (SF) and some of them were non-motile. Therefore, they could be misidentified as STEC SF O157:NM if proper molecular tests are not performed (Feng et al., 2010). For some draft genomes obtained from NCBI, prediction of the O157 serotyping gene *wzx* was difficult probably because of the breaking of contigs in the middle of the gene sequence, therefore a lower threshold of the percentage of minimum overlapping gene length was applied to confirm the genoserotype.

Intimin types of the isolates correlated to their genoserotypes; four of the genomes did not possess intimin and therefore were not considered to be EPEC. Our phylogenetic analysis based on cgMLST confirmed that *E. coli* O157:H7 belong to a different lineage than the non-H7 group, the latter belonging to diverse phylogenetic lineages (Sanjar et al., 2015). Our results support previous findings that horizontal transfer of the O157 antigen gene cluster has occurred independently among different *E. coli* lineages (Iguchi et al., 2011). Interestingly, isolates of O157:H45 clustered together independent of being a tEPEC or aEPEC. However, strain C844-97 was previously described to have lost its EAF plasmid (Hazen et al., 2013), and this may also be the case for strain C639-08 explaining their aEPEC features in spite of clustering together with tEPEC strains. Although the whole *bfp* gene cluster was present in tEPEC strains RN587/1 and ARS4.2123, they did not contain the complete EAF plasmid.

Almost all of the O157:H39 isolates included in this study belonged to recently evolved STs (e.g., ST4554 and ST5611). Alternatively, they were not reported previously, because no further characterization was performed as they did not carry the *stx* gene. We observed the presence of several plasmids, insertion elements and PAIs in O157:H39 isolates contributing to their virulence and resistance features. Three of the O157:H39 isolates of the new sequence type ST4554, shared an identical core genome but no epidemiological link could be identified among the patients from whom they were isolated.

The distribution of virulence genes almost perfectly correlated to the STs although slight intra-ST variations in virulence patterns were observed. The virulence genes were often carried on plasmids, PAIs, prophages, and insertion elements suggesting that these virulence factors were acquired from numerous sources via MGE, found specifically in the genome of pathogenic strains (Donnenberg and Whittam, 2001). The lineage specific acquisition of specific combinations of virulence factors may confer selective advantages contributing to the fitness of the pathogens favoring their establishment and transmission as new virulent clones (Reid et al., 2000). It was observed that ten of the O157:H16 isolates of ST10 (including one isolate from the US, one non-human isolate from France, and the rest from the Netherlands) shared almost identical virulence properties and were closely related in cgMLST with a maximum of a 41-allele difference (data not shown). A similar finding was observed in a previous study where O157:H16 strains were described as representatives of a widespread clone (Feng et al., 2010). However, the other two O157:H16 isolates, 3006 and Santai, belonged to two different STs, ST5502, and ST1011, respectively, and possessed almost no virulence genes. They were not classified as EPEC supporting the idea that the O and H serotyping might not represent the virulence status of the isolates and that the distribution of O and H antigen encoding genes is irrespective of the *E. coli* pathotype (Blank et al., 2003). Moreover, aEPEC isolates analyzed in this study were distributed in two completely different phylogenetic branches; one containing O157:H39 and O157:H45 (closer related to tEPEC) and another containing O157:H16 and O157:H26. This may be explained by horizontal transfer of the LEE PAI to different *E. coli* groups contributing to their EPEC feature (Feng et al., 2010).

Strain Santai belonging to the phylogenetic group D as does STEC O157:H7, appears to be more a multidrug resistant strain than a virulent one. The resistance genes were integrated into the chromosome of this organism through insertion elements and transposons from different species. EPEC 2827 was also resistant to several antibiotics and carried the drug resistance genes on a plasmid very similar to that of *S. enterica* (Hooton et al., 2014) and on an insertion element similar to a class1 integron of *Proteus* sp. (Guo et al., 2011). Isolates of ST4554 carried a plasmid similar to pCER1, a small, globally disseminated plasmid harboring sulphonamide and streptomycin resistance genes (Anantham and Hall, 2012). EPEC 1316 carried the resistance genes on a plasmid similar to pVR50A which also carries a *tra* gene cluster encoding conjugal transfer proteins (Beatson et al., 2015). Clearly, the transmission of MGEs plays a role in the evolution of new MDR lineages (Beceiro et al., 2013; Punina et al., 2015). EPEC infections can normally be recovered without antimicrobial therapy, therefore, the persistence of resistant EPEC strains is more likely to be related to selective pressure from antimicrobials applied at the population level or present in the environment (Scaletsky et al., 2010). Indeed the presence of resistance genes in *E coli* that originated from the environment have been described before (Hamelin et al., 2007).

In conclusion, *E. coli* O157:non-H7 isolates have evolved from multiple genetic backgrounds. They are distantly related to the most virulent EHEC clonal lineage EHEC O157:H7. Diversity in virulence and antibiotic resistance patterns have been observed among these O157:non-H7 isolates and mobile elements carried by these organisms could play a role in the evolution and dissemination of these virulent and resistant clones.

## Author contributions

Study design: MF, JR, AF. Performing Experiments: MF. Acquisition of data: MF, AK, JR. Analysis and interpretation of data: MF, KZ, JR, and AF. Drafting of manuscript: MF, KZ, AK, JR, and AF. Critical revision: MF, KZ, AK, JR, and AF.

## Funding

This study was partly supported by the Interreg IVa-funded projects EurSafety Heath-net (III-1-02 = 73) and SafeGuard (III-2-03 = 025).

### Conflict of interest statement

The authors declare that the research was conducted in the absence of any commercial or financial relationships that could be construed as a potential conflict of interest.
