# Supplementary material for: Virulence, Antimicrobial Resistance Properties and Phylogenetic Background of Non-H7 Enteropathogenic Escherichia coli O157
Source: Front Microbiol. 2016 Sep 28;7:1540. doi: 10.3389/fmicb.2016.01540 (PMC5039186; doi:10.3389/fmicb.2016.01540)
Supplement: Supplementary file 1 [file Image1.pdf]

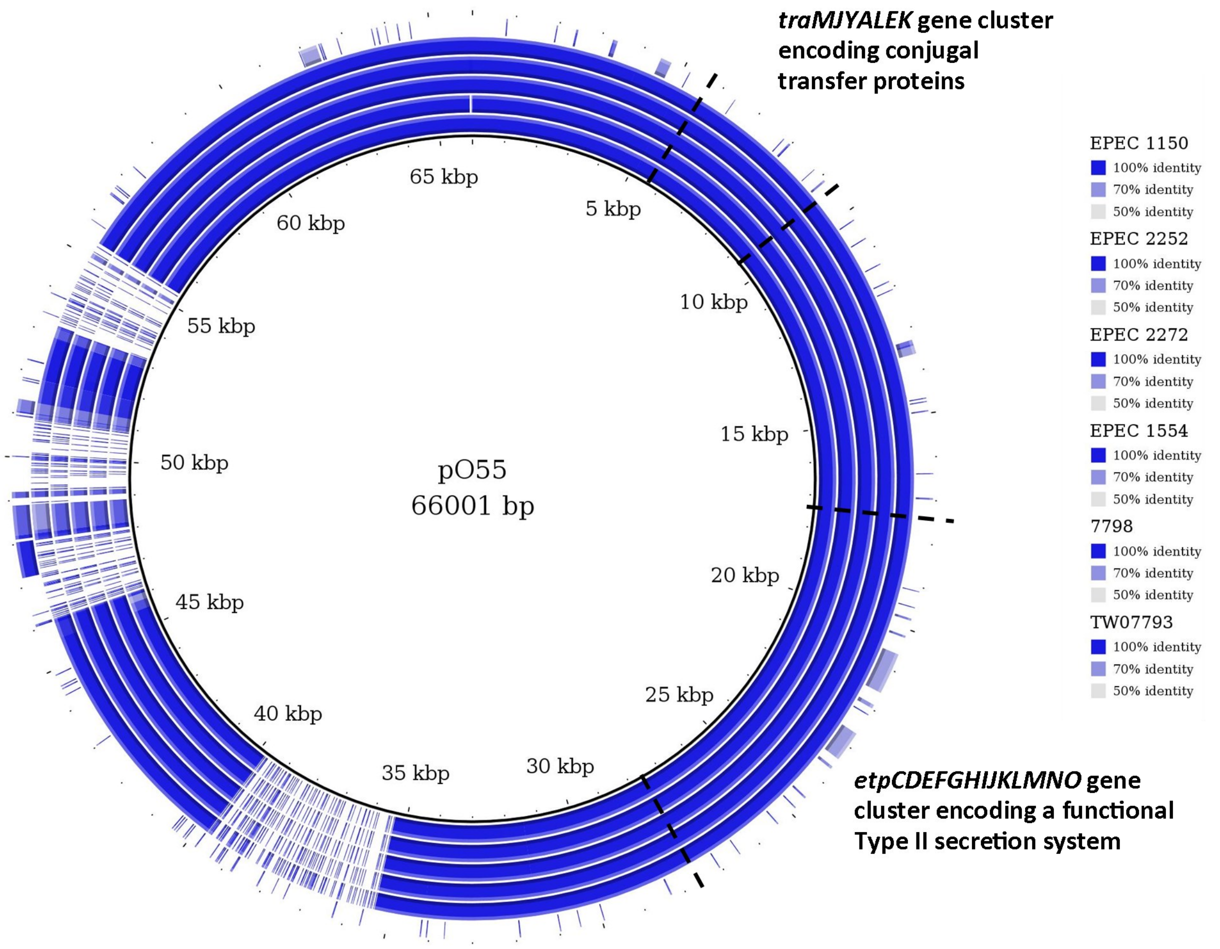

Supplementary Figure S1. pO55 like plasmid in O157:H39 Isolates. The figure shows BLAST comparison of the isolates against the reference plasmid pO55 of strain CB9615 (core black circle). Each ring represents one isolate; the gradients (dark, pale and white) of the color represent the sequence similarity (from 100% to 0%) between samples and reference. The order of the rings (from inner to outer) with the color gradient for sequence identity are shown in the legend (right).
