# Supplementary material for: Virulence, Antimicrobial Resistance Properties and Phylogenetic Background of Non-H7 Enteropathogenic Escherichia coli O157
Source: Front Microbiol. 2016 Sep 28;7:1540. doi: 10.3389/fmicb.2016.01540 (PMC5039186; doi:10.3389/fmicb.2016.01540)
Supplement: Supplementary file 2 [file Image2.PDF]

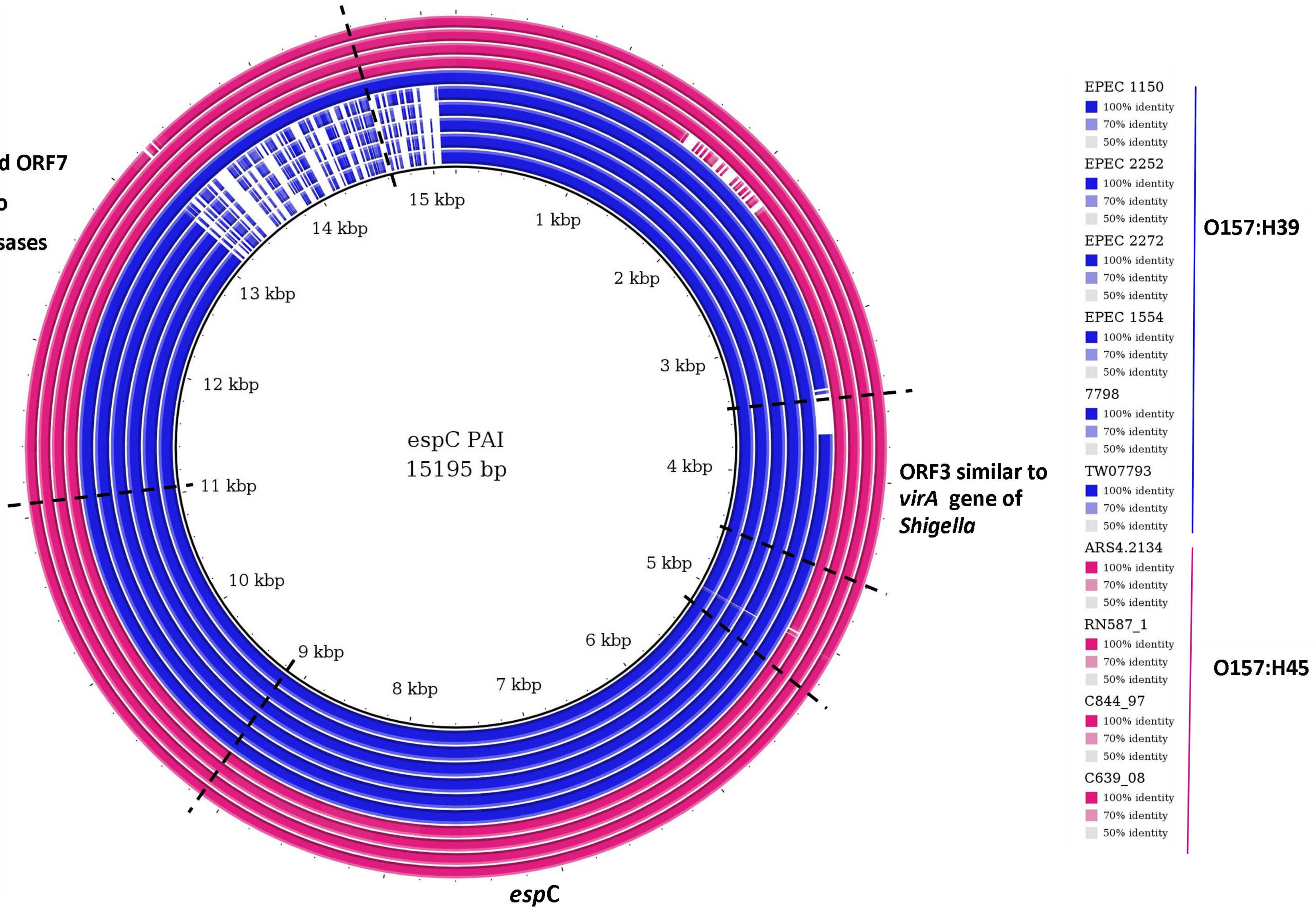

Supplementary Figure S2. ***espC*** **pathogenicity island in O157:H39 and O157:H45 isolates.** The figure shows BLAST comparison of the isolates against the reference *esp*C pathogenicity island of strain E2348/69 (core black circle). Each ring represents one isolate, different colors of the rings represent different genoserotypes. The gradients (dark, pale and white) of each color represent the sequence similarity (from 100% to 0%) between samples and reference. The order of the rings (from inner to outer) with the color gradient for sequence identity are shown in the legend (right).
